# Printing Smart Designs of Light Emitting Devices with Maintained Textile Properties †

**DOI:** 10.3390/ma11020290

**Published:** 2018-02-13

**Authors:** Inge Verboven, Jeroen Stryckers, Viktorija Mecnika, Glen Vandevenne, Manoj Jose, Wim Deferme

**Affiliations:** 1 Institute for Materials Research (IMO-IMOMEC)—Engineering Materials and Applications, Hasselt University, Wetenschapspark 1, 3590 Diepenbeek, Belgium; Inge.verboven@uhasselt.be (I.V.); stryckersjeroen@gmail.com (J.S.); Glen.vandevenne@uhasselt.be (G.V.); Manoj.jose@uhasselt.be (M.J.); 2 Interuniversity MicroElectronics Center (IMEC), IMOMEC, Universitaire Campus—Wetenschapspark 1, 3590 Diepenbeek, Belgium; 3 Institute of Textile Technology of RWTH Aachen, Otto Blumenthal Strasse 1, 52074 Aachen, Germany; viktorija.Mecnika@gerster.com; 4 Institute for Design Technology, Riga Technical University, Kalku Street 1, LV-1658 Riga, Latvia; 5 Flanders Make vzw, Oude Diestersebaan 133, B-3920 Lommel, Belgium

**Keywords:** electroluminescence, OLED, printing, textiles

## Abstract

To maintain typical textile properties, smart designs of light emitting devices are printed directly onto textile substrates. A first approach shows improved designs for alternating current powder electroluminescence (ACPEL) devices. A configuration with the following build-up, starting from the textile substrate, was applied using the screen printing technique: silver (10 µm)/barium titanate (10 µm)/zinc-oxide (10 µm) and poly(3,4-ethylenedioxythiophene)poly(styrenesulfonate) (10 µm). Textile properties such as flexibility, drapability and air permeability are preserved by implementing a pixel-like design of the printed layers. Another route is the application of organic light emitting devices (OLEDs) fabricated out of following layers, also starting from the textile substrate: polyurethane or acrylate (10–20 µm) as smoothing layer/silver (200 nm)/poly(3,4-ethylenedioxythiophene)poly(styrenesulfonate) (35 nm)/super yellow (80 nm)/calcium/aluminum (12/17 nm). Their very thin nm-range layer thickness, preserving the flexibility and drapability of the substrate, and their low working voltage, makes these devices the possible future in light-emitting wearables.

## 1. Introduction

Smart luminous textiles are of great interest for applications such as clothing, interior design and visual merchandizing. Moreover, luminous textiles are beneficial for protective clothing and sportswear in order to improve safety by a higher visibility and interactive design for non-verbal communication. Additionally, luminous textiles have potentials for healthcare and medicine applications such as phototherapy.

At present, such smart textiles are mostly limited to the integration of light emitting devices (LED) or optical fibres [1]. This approach however is limited to small-scale luminous textiles. An optional solution for the implementation of large-scale luminous surfaces on textiles is brought by applying printing technologies. There are a number of research projects that have investigated scenarios to incorporate light-emitting devices on textiles [2,3]. These address alternating current powder electroluminescence (ACPEL) and organic light emitting diodes (OLED) technology. Nevertheless, these are mostly limited to printing luminous structures on non-textile substrates and subsequently integrating them onto textile surfaces.

This work addresses two different approaches implemented directly on textiles substrates: improved screen-printed designs of ACPEL devices and direct deposition of OLEDs. Diverse smart designs of the ACPEL devices were suggested to preserve textile properties such as flexibility, drapability and air permeability. The complete layer stack silver (Ag) (10 µm)/barium titanate (BaTiO_3_) (10 µm)/zinc-oxide (ZnO) (10 µm)/poly(3,4-ethylenedioxythiophene)poly(styrenesulfonate) (PEDOT:PSS) (10 µm) was applied using the screen printing technique. A pixel-like design of the printed layers was selected and different geometries were implemented. In order to comprehend the interaction between the textile substrate, the applied functional layers and the selected design, the samples were tested on flexibility, air permeability and light output and their morphology after mechanical stress was investigated. Organic light-emitting devices (OLED) are more challenging to apply directly on textiles, but promise to be the future in light-emitting wearables. These devices were build out of the following layers: polyurethane (PU) or acrylate (10–20 µm) as the smoothing layer/Ag (200 nm)/PEDOT:PSS (35 nm)/super yellow (80 nm)/calcium/aluminum (Ca/Al) (12/17 nm) ending up with a device stack of maximum 0.5 µm and therefore maintaining the flexibility and drapability of the textile substrate. Due to the roughness of the textile substrate a planarizing layer of polyurethane (PU) or acrylate (10–20 µm) had to be applied directly on the substrate before completing the rest of the stack. OLEDs have a high brightness and a low power consumption. To protect these devices from fast degradation from contact with oxygen or water vapour, an encapsulation layer is necessary.

## 2. Results

### 2.1. ACPEL Devices

**Figure 1:**
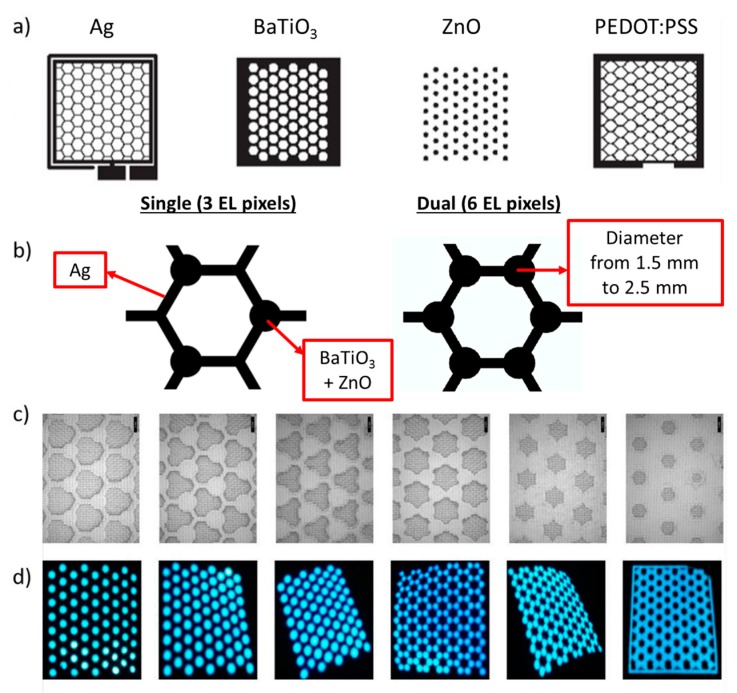
(**a**) Design of the single layers of the ACPEL (alternating current powder electroluminescence) build-up; (**b**) a zoom on a hexagon cell, showing clearly the applied size and number of the pixels; (**c**) SEM (Scanning electron microscopy) micrographs of the different printed ACPEL devices; (**d**) images of the light-emitting area of the ACPEL stacks.

Literature shows that ACPEL devices can be printed on a variety of substrates [4]. The thickness of the complete device is about 40 µm and mostly it is applied as full area coverage. However, this will mask the benefits of the textile such as air permeability and drapability. Therefore, in this work, a special design, based on a hexagonal cell structure is proposed. The design of the stack can be seen in Figure 1a.

Both the bottom layer (Ag) and the top layer (PEDOT:PSS) are screen printed in this honeycomb structure. The line width is 0.5 mm and both layers are deposited in such a way that they are not touching, to prevent electrical shorts. The dielectric layer and the light emitting layer consist of 1.5 to 2.5 mm pixels. They can be printed on each crossing of the hexagon structure or on half of them. By changing the diameter and the number of pixels per hexagon, the light emission, but also the air permeability and the crease recovery, can be adapted. A schematic view of a hexagon cell structure is depicted in Figure 1b. Scanning electron microscopy (SEM) is applied to look at the final printed ACPEL device in detail. In Figure 1c, SEM images of the ACPEL devices printed on the polyester textile are shown. From left to right, the diameter of the pixels is changed from 1.5 mm over 2 mm up to 2.5 mm. This is the case for the first three images from the left, where only three pixels are printed in one hexagon cell. Also, for the last three SEM images, this change of diameter is applied but now six pixels are printed in one hexagon cell. It is clear from these SEM micrographs that the area of uncoated textile changes (dark grey area) when altering the diameter and the number of pixels per cell. In Figure 1d, the light emission can be noted. Due to the design of the ACPEL stack, only the pixels show light emission.

**Figure 2:**
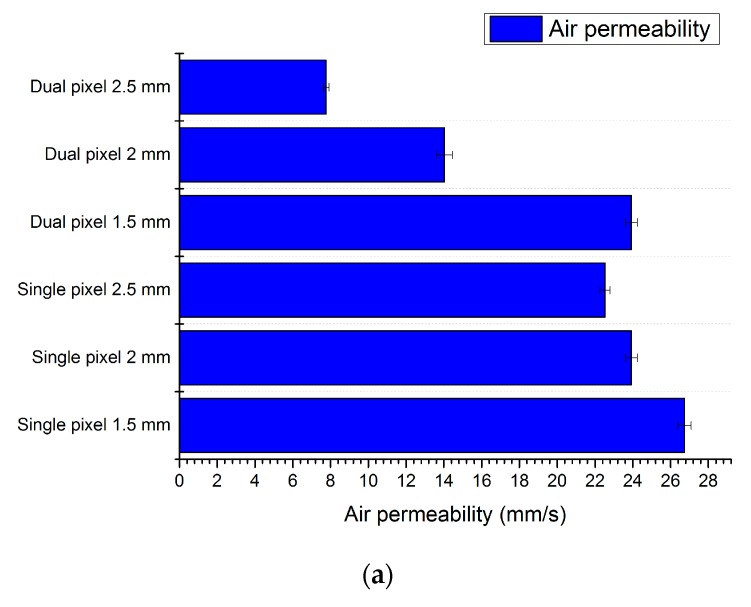

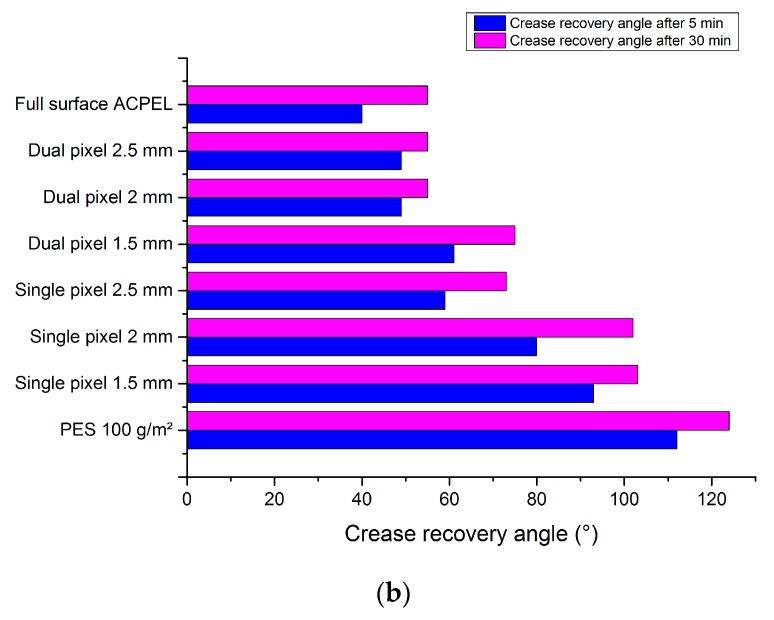
The air permeability (**a**) and the crease recovery angle (**b**) of the screen printed ACPEL devices on polyester textile substrates.

Finally, Figure 2 indicates the influence of the design on the properties of the textile substrate after screen printing the ACPEL device.

**Figure 3:**
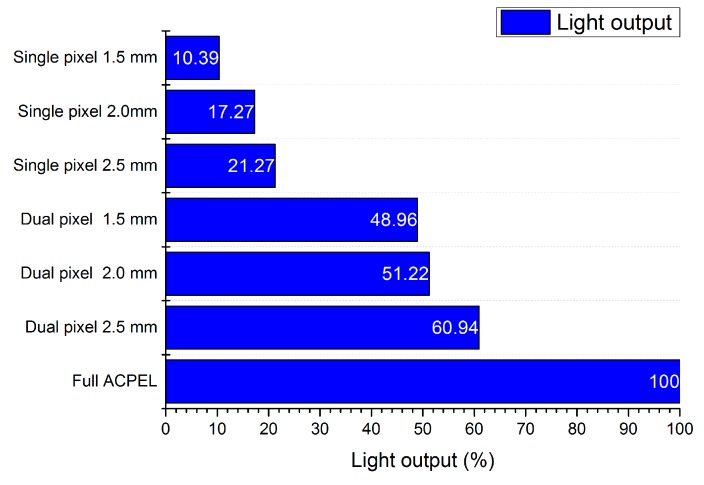
Light output of the ACPEL devices.

In Figure 2a, the air permeability, as measured by a FX3300 LabAir IV Air Permeability tester (Textest AG, Schwerzenbach, Switzerland), for the different designs is shown. It is clear from this graph that more air can pass if the diameter of the pixels is smaller. This is logical, of course, as less textile surface is covered. The difference between the single pixel structure, where only three pixels are printed per hexagon call, for the different diameters is however not as big as for the dual pixel structure, with six pixels per hexagon cell. It can also be noted that the difference between the dual pixel structure, with a diameter of 1.5 mm and the single pixel structure, with a pixel diameter of 2.5 mm and 2 mm, is comparable. This graph is in correspondence with the calculated area coverage of the textile substrate. In Figure 2b one can see the crease recovery measurements. In this experiment, the textile is folded and kept as such for 5 min and for 30 min by applying a weight of 1 kg on top of the double folded textile. After these 5 or 30 min, the weight is removed and it is recorded how far the textile will reverse back to its initial state. This is denoted as the crease recovery. From the figure it can be seen that, in comparison to an uncoated polyester substrate (last line of the graph), the crease recovery was smaller for all samples. However, in comparison with ACPEL devices printed as a full covering on the polyester textile, the crease recovery, especially for the single pixel structures, is very good. The light output performances were acquired using a Keithley 2401 (Keithley, Cleveland, OH, USA) source to measure the current and voltage characteristics and an absolute calibrated integrating sphere spectrometer from Avantes to determine the irradiance per wavelength [5]. The light output was obtained by comparing the electrical power to the coated area. In Figure 3 the light output of the different designs are compared to that of a fully covered ACPEL device. This demonstrates that the light output is halved when a dual pixel design is used instead of a fully covered surface. The light output of a device with the single pixel design is even less than one fourth of that of a fully covered device. 

Based on these experiments and on the light emission measurements, which can be found in [5], the single pixel structure with a diameter of 2 mm is seen as the most optimal, when reserving the textile properties has priority. When, however, the light output is of high importance, the best option can be found in the dual pixel design with a diameter of 1.5 mm.

### 2.2. OLED Devices

**Figure 4:**
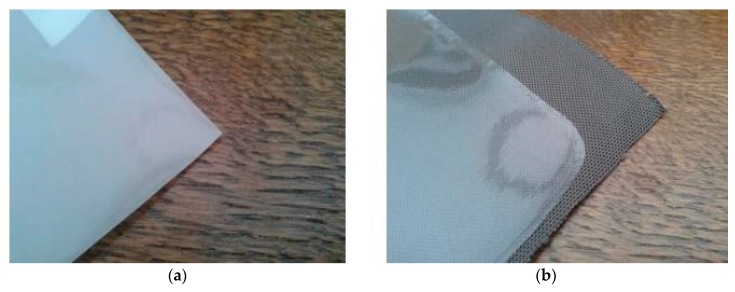
The textile substrate covered with PU (polyurethane) (**a**) or acrylate (**b**) to smoothen the textile roughness towards nm-range.

Applying OLEDs to textiles is not that straightforward as is the case for the screen-printed ACPEL devices discussed above. First of all, the total thickness of the OLED stack is only 0.5 µm, which is even smaller than the roughness of the underlying textile substrate. Further, the deposition techniques to do so are not as standard as the screen printing technique from above. The advantages of using OLEDs, however, are numerous. Since they are made of very thin nm layers, the devices can be applied to flexible substrates. The emitted light has a high brightness, a uniform light output and a wide range of vision. OLEDs require a low power supply (3–5 V), have a low energy consumption and a good efficacy. Important disadvantages or challenges, however, have to be taken into account. The devices degrade very quickly due to water vapour and oxygen. Therefore water vapour transmission rates (WVTR) and oxygen transmission rates (OTR) must be lower than respectively 10^−6^ g·m^−2^ per day and 10^−3^ cm^3^·m^−2^ per day, indicating a very high barrier layer is necessary [6]. Some of the applied techniques to deposit the OLED layers are very expensive and not roll-to-roll compatible. However, more and more less expensive and roll-to-roll compatible printing techniques are emerging. These other deposition techniques and the OLED stack to be applied to textiles will be discussed in more detail in this part of the paper. As mentioned, the surface of the textile substrate is quite rough (µm-range) compared to the nm-range layer thickness of the OLEDs. This roughness can be ruled out by the deposition of a planarizing or covering layer. Printable PU or acrylate are therefore laminated on top of the textile substrates as is shown in Figure 4, with a thickness between 10 and 20 µm to bring the micrometer roughness of the textile substrate to a nanometer roughness.

**Figure 5:**
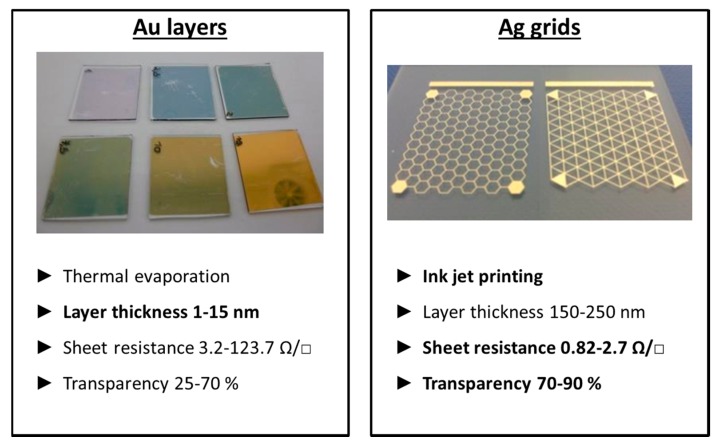
The transparent top electrode can be deposited by applying a full covering Au layer or by inkjet printing Ag grids.

As previously stated, OLEDs degrade immediately in ambient conditions, making good encapsulation indispensable. Therefore a transparent barrier layer is applied using plasma techniques. A first oxygen-free silicon nitride (SiN) base layer serves as a protection for later depositions. This layer is followed by an alternating system of high barrier inorganic materials (such as silicon oxide (SiOx)) and a softer, low barrier organic materials. This barrier system brings a halt to defect formation and subsequently increases the diffusion length and the barrier properties. More information on the topic of encapsulation can be found in [7]. The bottom electrode or anode is a thermally evaporated silver (Ag) layer of 200 nm. Subsequently the hole injection/transport layer PEDOT PSS, a polymer mixture, is spin coated to obtain a 35 nm film. As an active layer, the PPV polymer Super Yellow is used to spin coat a layer of 80 nm inside an inert atmosphere glovebox system. Both the lab-scaled spin coating and thermal evaporation technique can be replaced by inkjet printing and ultrasonic spray coating. Inkjet printing is a contactless printing process where a digital image is recreated by ejecting ink droplets onto a substrate. The large-area deposition technique ultrasonic spray coating forms layers by atomizing the ink at the nozzle of the spray head into a continuous flow of micro sized spherical droplets. Both techniques are less expensive and roll-to-roll compatible. It was shown in earlier work of the authors [8] that the active light-emitting layer can be ultrasonically spray coated without changing or damaging the polymer side-chain or backbone of the PPV polymer. As the textile substrate is not transparent, a top emitting polymer OLED (TEOLED) is prepared where the photons have to escape the device through the top transparent electrode or cathode. To obtain a transparent cathode, two different methods are tested in this work, i.e., applying printed metal grids or evaporating very thin metal layers. For comparison, inkjet-printed Ag grids and very thin thermally evaporated golden (Au) layers were assessed by their transparency and sheet resistance. Hexagonal and triangular shaped Ag grids were inkjet-printed on glass substrates with a thickness of 150–250 nm. They showed a low sheet resistance of 0.82–2.7 Ω/□ and a high transparency of 70–90%. Very thin and completely covering Au layers of 1–15 nm were thermally evaporated on glass substrates. Here a higher sheet resistance of 3.2–123.7 Ω/□ and a lower transparency between 25–70% was found. An overview of these results can be seen in Figure 5.

**Figure 6:**
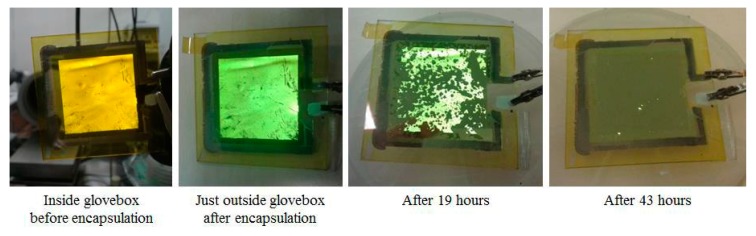
The OLED (organic light emitting diodes) after before and after encapsulation showing a fast degradation with full fading after only 43 h.

Considering only these two characteristics, the Ag grids score much better on both as can also be found back in earlier work of the authors [9]. However, the used commercially available Ag ink has to be sintered at a temperature of 200 °C, which will destroy all underlying layers. New Ag inks, based on precursors rather than on Ag nanoparticles, are now available with a considerable lower sintering temperature [10] and therefore, applying this grid structure is the most promising. A low work function material, such as calcium (Ca), has to be used in between the light emitting layer and the top Ag layer to align the energy levels for the proper functioning of the OLED. This material is usually thermally evaporated. Therefore, at this time, preference was given to a thermally-evaporated Ca/Ag cathode of respectively 12 and 17 nm. In this work, the complete OLED stack was deposited on glass, PET and textile substrates. The encapsulated glass OLED sample had some visual defects and pinholes, as can be seen in Figure 6. After applying the barrier layer, the OLED sample was taken out of the glovebox system to investigate the effects of ambient conditions on the encapsulation. After 19 h the OLED had already lost more than half of its light output and after 43 h only a few luminous pixels were visible. This shows that applying the barrier layer is a promising encapsulation strategy, but more research is needed to improve the OLED’s characteristics and lifetime.

**Figure 7:**
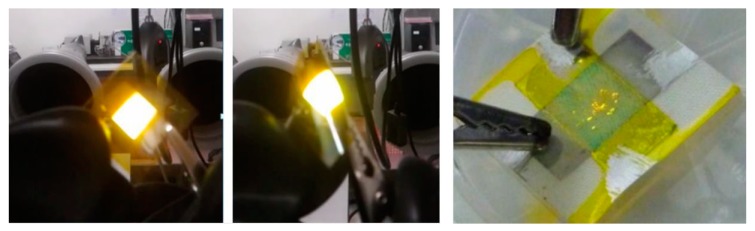
From left to right: OLED on PET (polyethylene terephthalate) just after deposition; OLED on PET showing the stable light emission even during flexing and bending; OLED stack printed on textile.

Finally, the OLED stack has been deposited onto PET foil and textiles. The OLED on PET had a nice uniform light output and could be bent without any output loss or cracks in the layers, displaying the flexibility of the OLED device as shown in Figure 7. However, for the textile-based OLED, only a few luminous pixels could be distinguished. The reasoning behind this bad light emission for the textile-based OLED is that this device employed printable PU as planarizing layer. This PU layer was effected by the chlorobenzene used as solvent for the light emitting polymer Super Yellow. Consequently a lot of defects were introduced into the OLED stack, making an informal light output impossible.

## 3. Discussion and Conclusions

It has been shown in this work that light emitting devices can be printed on textile substrates applying different designs and different printing and coating techniques. First of all, an ACPEL device is fully screen-printed in an adapted, smart design such that the breathability and the drapability of the textile substrate are enhanced. It was shown that adapting the design (diameter and number of pixels per hexagon cell) can influence the air permeability and the crease recovery. The application of OLEDs on textiles shows several advantages, being very thin and flexible devices with a low power supply, low energy consumption, a good efficacy, a bright and uniform light output and a wide range of vision. Nevertheless, the usage of a high barrier layer is necessary and some applied deposition techniques are not roll-to-roll compatible and quite expensive. At the moment high barrier layers are still applied using a combination of printing and plasma techniques, but for the actual OLED stack layers alternative techniques have been pushed forward, such as inkjet printing and ultrasonic spray coating. Adequate research into a proper barrier layer, a planarizing layer, a transparent top electrode and roll-to-roll deposition techniques is ongoing and will bring the OLED technology from the class of PET foils towards textile substrates. The combination of both results presented in this paper can finally lead to a pixelated OLED structure on textile substrates for enhanced light emission without hampering the textile properties.

## 4. Materials and Methods

**Figure 8:**
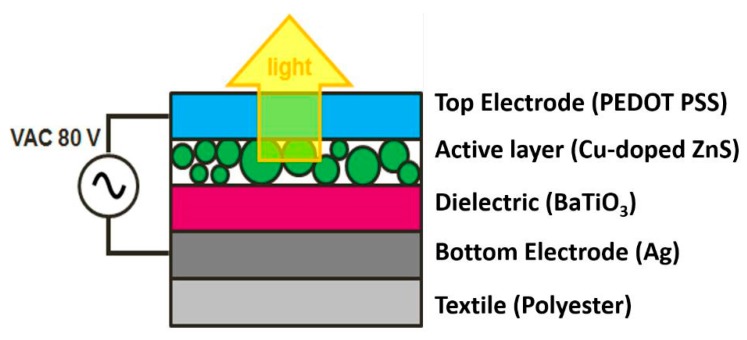
Build-up of the ACPEL technology.

As mentioned above two diverse technologies for lighting are examined on their printability on textile substrates. Figure 8 shows the typical layer build-up of an ACPEL device. All of the layers, with a thickness of 10 µm, are deposited on top of each other using the screen printing technique. The textile used in this work was a polyester woven fabric (100% PES—washed and fixated—kw11401 from Concordia Textiles (Valmontheim, Belgium) with a roughness average Ra of 6 µm. The first Ag layer (from Gwent) fulfils a dual purpose, as a bottom electrode and as a planarizing layer. This layer is followed by a dielectric layer (BaTiO3 from Gwent, Pontypool, United Kingdom) and a light emitting layer (Cu-doped ZnS from Gwent, Pontypool, United Kingdom). They are stacked in between two electrodes and therefore, a capacitor build-up is achieved. A transparent top-electrode (PEDOT:PSS EL-P 3145 ink from Orgacon, Mortsel, Belgium) completes the stack. After screen printing each layer, they are subsequently thermally annealed at 130 °C for 10 to 30 min. When a AC voltage of 80 V is applied with a frequency of 400 Hz, light is generated and coupled out through the transparent top-electrode. 

**Figure 9:**
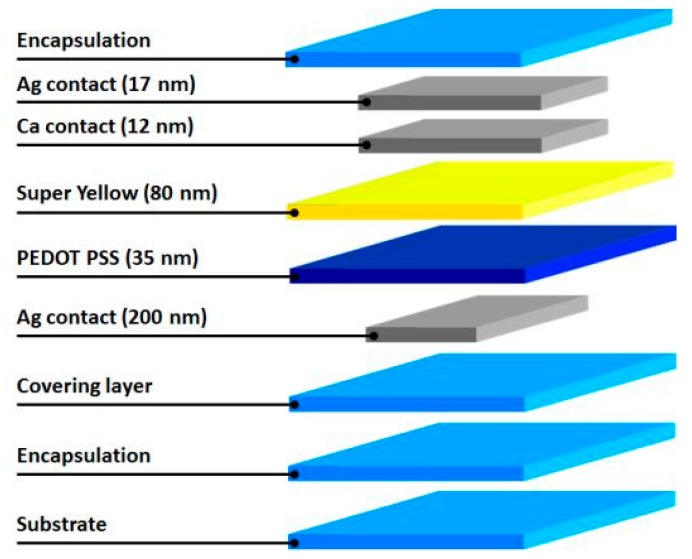
Build-up of the OLED device.

**Figure 10:**
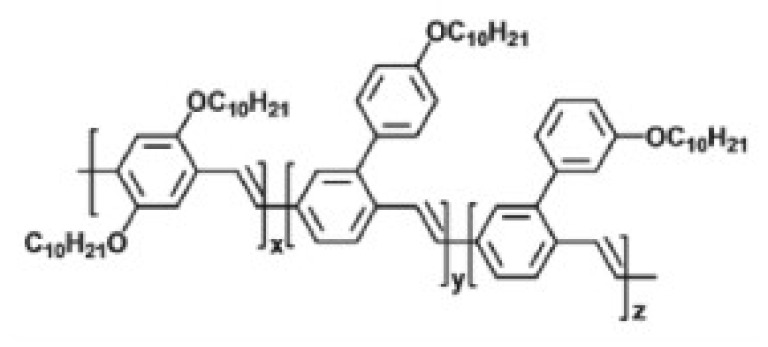
Molecular structure Super Yellow [11].

For the second approach, organic light-emitting diodes (OLED) are deposited. The TEOLED stack (Figure 9) is produced by implementing different deposition techniques to apply the layers on glass, polyethylene terephthalate (PET) and textile substrates. Due to the relatively high surface roughness of the textile substrates, an additional planarizing/covering layer is required. To equalize the surface polyurethane (PU) or acrylate is laminated onto the textile substrate with a thickness between 10–20 µm. By applying plasma techniques, a barrier layer, composed out of a stack of alternating organic and inorganic layers with a total thickness of 1 µm, was added on top of the substrate or planarizing layer. Afterwards, an Ag anode of 200 nm is thermally evaporated at a base pressure of 10^−7^ mbar. Under a fume hood a hole injection/transport layer PEDOT PSS (Clevios™ P AI 4083 from Heraeus, Hanau, Germany) of 35 nm is spin coated. As an active layer the PPV–polymer super yellow (PDY-132 from Merck, Darmstadt, Germany) (Figure 10) was dissolved in chlorobenzene with a mass concentration of 5 mg/mL and stirred overnight at 50 °C. A layer of 80 nm was spin-coated in an inert atmosphere glovebox system (O_2_/H_2_O ppm <0.1). Subsequently a transparent cathode of Ca and Ag was thermally evaporated at a base pressure of 10^−7^ mbar with a thickness of respectively 12 and 17 nm. The stack is completed with another barrier layer deposited by plasma techniques.

## References

[1] Peters K. (2011). Polymer optical fiber sensors: A review. Smart Mater. Struct..

[2] Linz T., von Krshiwoblozki M., Walter H., Foerster P. (2012). Contacting electronics to fabric circuits with non-conductive adhesive bonding. J. Text. Inst..

[3] Kim W., Kwon S., Lee S.-M., Kim J.Y., Han Y., Kim E., Choi K.C., Park S., Park B.C. (2013). Soft fabric-based flexible organic light emitting diodes. Org. Electron..

[4] Li Y., Tan L., Hao X., Ong K.S., Zhu F., Hung L. (2005). Flexible top-emitting electroluminescent devices on polyethylene terephthalate substrates. Appl. Phys. Lett..

[5] Stryckers J., Mecnika V., Vangever T., Vandevenne G., Verboven I., Nagels S., Jose M., Manca J., Hoerr M., Scheulen K. (2018). Light Emitting Textile: Printing innovative electroluminescent designs to maintain the textile properties. Mater. Des..

[6] Wuu D.S., Chen T.N., Wu C.C., Chiang C.C., Chen Y.P., Horng R.H., Juang F.S. (2006). Transparent Barrier Coatings for Flexible Organic Light-Emitting Diode Applications. Chem. Vapor Depos..

[7] Troia M., Leins M., Schulz A., Walker M., Deferme W., Govaert F., Hoerr M., Mecnika V., Van Parys M., Hirth T. Oxygen barrier layers for flexible OLED devices. Proceedings of the 17th Fachtagung für Plasmatechnologie.

[8] Gilissen K., Stryckers J., Verstappen P., Drijkoningen J., Heintges G.H.L., Lutsen L., Manca J., Maes W., Deferme W. (2015). Ultrasonic spray coating as deposition technique for the light-emitting layer in polymer LEDs. Org. Electron..

[9] Vandevenne G., Marchal W., Verboven I., Drijkoningen J., D’Haen J., Van Bael M.K., Hardy A., Deferme W. (2016). A study on the thermal sintering process of silver nanoparticle inkjet inks to achieve smooth and highly conducting silver layers. Phys. Status Sol. (a).

[10] Marchal W., Vandevenne G., D’Haen J., de Andrade Almeida A.C., Sola M.A., van den Ham J., Drijkoningen J., Elen K., Deferme W., Van Bael M.K. (2017). Ultrasonically spray coated silver layers from designed precursor inks for flexible electronics. Nanotechnology.

[11] Gambino S., Bansal A.K., Samuel I.D. (2010). Comparison of hole mobility in thick and thin films of a conjugated polymer. Org. Electron..

